# Compressed sensing to accelerate magnetic resonance spectroscopic imaging: evaluation and application to ^23^Na-imaging of mouse hearts

**DOI:** 10.1186/s12968-015-0149-6

**Published:** 2015-06-15

**Authors:** Mahon L. Maguire, Sairam Geethanath, Craig A. Lygate, Vikram D. Kodibagkar, Jürgen E. Schneider

**Affiliations:** British Heart Foundation Experimental Magnetic Resonance Unit, Radcliffe Department of Medicine – Division of Cardiovascular Medicine, University of Oxford, Roosevelt Drive, Oxford, OX3 7BN UK; Medical Imaging Research Centre, Dayananda Sagar College of Engineering, Bangalore, 560078 India; School of Biological and Health Systems Engineering, Arizona State University, Tempe, AZ 85287-9709 USA

**Keywords:** Compressed sensing, Magnetic resonance spectroscopic imaging, Chemical shift imaging, Mouse, Sodium

## Abstract

**Background:**

Magnetic Resonance Spectroscopic Imaging (MRSI) has wide applicability for non-invasive biochemical assessment in clinical and pre-clinical applications but suffers from long scan times. Compressed sensing (CS) has been successfully applied to clinical ^1^H MRSI, however a detailed evaluation of CS for conventional chemical shift imaging is lacking. Here we evaluate the performance of CS accelerated MRSI, and specifically apply it to accelerate ^23^Na-MRSI on mouse hearts *in vivo* at 9.4 T.

**Methods:**

Synthetic phantom data representing a simplified section across a mouse thorax were used to evaluate the fidelity of the CS reconstruction for varying levels of under-sampling, resolution and signal-to-noise ratios (SNR). The amplitude of signals arising from within a compartment, and signal contamination arising from outside the compartment relative to noise-free Fourier-transformed (FT) data were determined. Simulation results were subsequently verified experimentally in phantoms and in three mouse hearts *in vivo*.

**Results:**

CS reconstructed MRSI data are scaled linearly relative to absolute signal intensities from the fully-sampled FT reconstructed case (R^2^ > 0.8, *p*-value < 0.001). Higher acceleration factors resulted in a denoising of the reconstructed spectra, but also in an increased blurring of compartment boundaries, particularly at lower spatial resolutions. Increasing resolution and SNR decreased cross-compartment contamination and yielded signal amplitudes closer to the FT data. Proof-of-concept high-resolution, 3-fold accelerated ^23^Na-amplitude maps of murine myocardium could be obtained within ~23 mins.

**Conclusions:**

Relative signal amplitudes (i.e. metabolite ratios) and absolute quantification of metabolite concentrations can be accurately determined with up to 5-fold under-sampled, CS-reconstructed MRSI. Although this work focused on murine cardiac ^23^Na-MRSI, the results are equally applicable to other nuclei and tissues (e.g. ^1^H MRSI in brain). Significant reduction in MRSI scan time will reduce the burden on the subject, increase scanner throughput, and may open new avenues for (pre-) clinical metabolic studies.

## Background

Magnetic Resonance Spectroscopic Imaging (MRSI) allows non-invasive investigation of regional metabolic processes *in vivo*, but suffers from long scan-times due to low metabolite concentrations, slow spatial encoding schemes, and low MR sensitivity (for nuclei other than protons). This versatile technique would benefit from a reduction in scan-time in order to make it more clinically applicable. Single- or multi-shot echo-planar spectroscopic imaging (EPSI) sequences [1-6] have been proposed to reduce MRSI scan-time. Indeed, Furuyama et al. have evaluated the performance of CS EPSI based 2D *J*-resolved spectroscopy [7,8]. However, EPSI is known to provide lower sensitivity compared to classical chemical shift imaging (CSI) [7]. Low metabolite concentrations and reduced MR sensitivity limit the applicability of parallel imaging. Conversely, Compressed Sensing (CS) is a technique for accelerating the inherently slow data acquisition process, and is well suited for MRSI due to its intrinsic denoising effect [9]. CS has been used to accelerate ^1^H- [10,11], hyperpolarized ^13^C- [12-14],^23^Na- [15], ^31^P-MRSI [16], and multi-dimensional NMR experiments [17,18]. The scan time reductions ranged from 2- [15,13] to 18-fold [14]. The application of CS to (pre-) clinical *in vivo* imaging necessitates thorough examination of the conditions under which it is robust. However, little systematic evaluation of the influence of SNR and spatial resolution on the achievable scan-time reductions and quantification of metabolite signals has been performed to date [7,13,19]. Whilst the work by Geethanath et al. [11] made an important step forward in the use of CS-MRSI, it generated some debate regarding the reliability of its use in the clinic [20,21]. We present a detailed performance evaluation of the CS-reconstruction developed by Geethanath et al. in the context of ^1^H-MRSI [11]. For the first time we then apply CS to ^23^Na-MRSI on mouse hearts demonstrating the potential of CS for high resolution spectroscopic imaging *in vivo*.

## Methods

### Synthetic phantom data

**Fig. 1:**
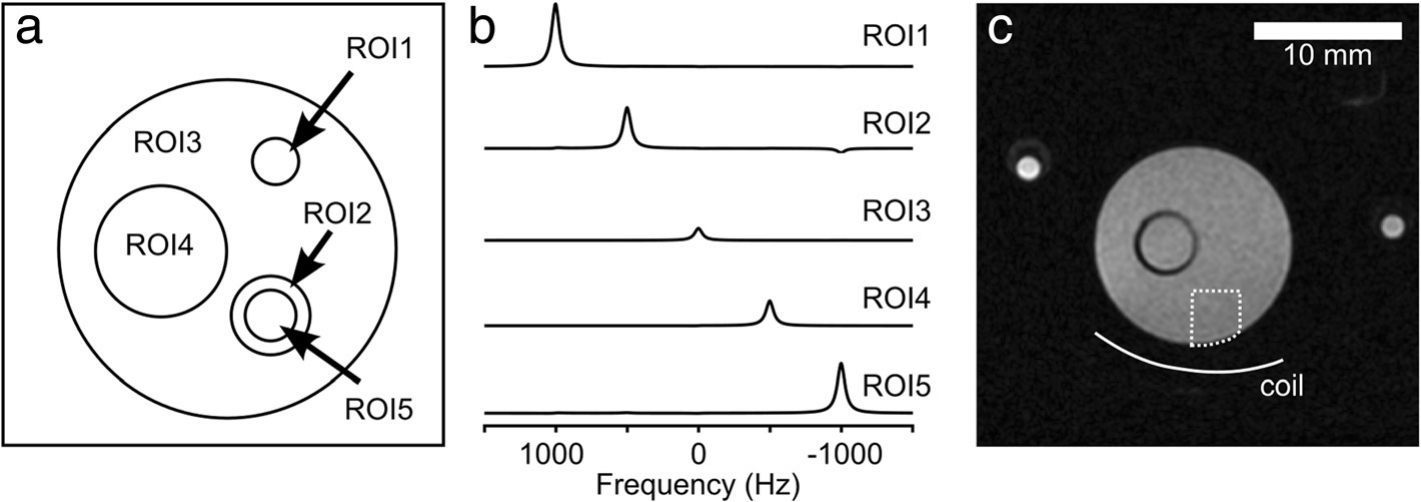
Layout of phantoms. **a** Layout of the virtual phantom. Five compartments were defined within the phantom representing aorta, myocardium, skeletal muscle, liver, and left ventricular blood pool (ROI1-5 respectively) with relative amplitudes of 5, 3, 1, 2, and 4. **b** The spectra corresponding to voxels within each compartment contain a single Lorentzian resonance at a unique frequency. Example spectra from each compartment within the virtual phantom, reconstructed at 64 × 64 voxels, are shown. Resonance frequencies for ROI1-5 were −1000, −500, 0, 500, 1000 Hz, respectively, with a T_2_^*^ of 50 ms. All spectra are plotted using the same axes. The small peak visible at −1000 Hz in ROI2 corresponds to signal contamination from ROI5. **c** ^1^H-MR image of the experimental phantom containing NaCl solutions (inner compartment: 20 mM NaCl; outer compartment: 100 mM NaCl). The two coaxial compartments on the left and right of the phantom contained 200 and 500 mM NaCl, respectively, and served as concentration references. The area used for data normalization is indicated by the dashed line (3.3 x 3.5 mm ROI), and the position of the surface coil used for ^23^Na signal reception is shown

An *in-silico* noise-free phantom containing five compartments and representing a simplified mouse thorax was constructed in the image domain (256 × 256 voxels). Each compartment contained a single Lorentzian resonance at a unique frequency (Fig. 1a,b). This enabled quantification of inter-compartment signal contamination arising from CS reconstruction as the spatial origin of the signal could be determined from its frequency. These data were Fourier transformed (FT) into the time domain and the central N × N *k*-space points (N = 16, 24, 32, 48, 64) taken as low-resolution representations. Gaussian noise was added to vary the SNR in the image domain (2, 4, 8, 16, 32, 64, 128, 256 and ∞ (i.e. noise-free)), where SNR was defined as the ratio of peak-height to the standard deviation of the noise of the real part of the spectrum.

### Hardware

All ^23^Na-MRSI data were acquired on a horizontal 9.4 T MR system equipped with a VnmrS Direct Drive2 console and 60 mm i.d. 1 T/m imaging gradients (Agilent Technologies, USA). An actively decoupled ^23^Na quadrature birdcage transmit resonator (i.d. 39 mm) was used in combination with a 14 mm square surface receive coil (Rapid Biomedical, Germany). ^1^H anatomical images were acquired using a 39 mm quadrature birdcage resonator (Rapid Biomedical, Germany).

### Phantom experiments

Validation experiments were conducted on a phantom comprised of two coaxial compartments with the inner compartment (i.d. 3.6 mm, volume ~ 1 ml) containing 20 mM NaCl, and the outer compartment (i.d. 13.5 mm, volume 15 ml) containing 100 mM NaCl (Fig. 1c). The phantom also contained two coaxial concentration references containing 200 and 500 mM NaCl, which, however, were not used for this study. Fully sampled, single average, 2D axial slice-selective MRSI data (60° flip angle, 30 × 30 mm FOV, 2 mm slice, 96 × 96 PE steps, TR/TE = 150/0.50 ms, 9216 FIDs, acquisition time ~24 mins) were acquired and repeated 32 times (total acquisition time: 12.8 h). The central N × N *k*-space points (N = 16, 24, 32, 48, 64, 96) from multiple repetitions were combined to give MRSI data sets with varying spatial resolution and SNR.

### *In vivo* experiments

Cardiac ^23^Na-MRSI experiments were conducted on three female wild-type C57BL/6 mice (body weight 27.5 ± 4.0 g). After induction of anaesthesia (4 % isoflurane in 100 % oxygen), mice were positioned prone in a dedicated animal cradle with the heart centred over the surface coil, and maintained at 37 °C and 1.5–2.0 % isoflurane in 2 L/min oxygen flow throughout the MRSI experiments. Cardiac and respiratory signals were continuously monitored using an in-house developed ECG and respiratory gating device [22]. All investigations were approved by the local ethical review committee and conformed to the Animals (Scientific Procedures) Act 1986 (UK) incorporating European directive 2010/63/EU.

Using the same MRSI sequence as for the phantom data, threefold prospectively under-sampled ^23^Na data were acquired in mouse hearts *in vivo* in short-axis orientation (FOV 30 × 30 mm, 64 × 64 PE steps, 2 mm slice thickness, 8 averages, 40° flip angle, cardiac triggered, TR/TE = ~125/0.85 ms (i.e. one cardiac cycle), total acquisition time ~23 mins). Under-sampling was carried out as described previously [11].

### Data analysis

All data were reconstructed and analysed in Matlab2013a (Mathworks, USA). Synthetic and acquired phantom data were retrospectively 1- to 5-fold under-sampled using a pseudo-random sampling scheme and reconstructed using a CS reconstruction described previously [11]. Signal amplitudes (i.e. peak maximum of the real component of the complex spectra), and contamination for CS reconstructed data and fully-sampled, FT reconstructed, data were compared. Signal amplitudes obtained from the CS reconstructed data were subject to linear regression analysis with the FT reconstructed fully-sampled data. A *p*-value of < 0.05 indicated a statistically significant correlation between the datasets. Signal contamination, defined as the total signal amplitude from resonances originating from outside the ROI detected within the ROI divided by the number of voxels in the ROI, was assessed for the synthetic phantom only. The ^23^Na myocardium/blood-ratio was estimated *in vivo* by calculating the mean ratio from six pairs of adjacent voxels located in the myocardium and the left ventricular blood-pool. Ratios are quoted as mean ± standard deviation. SNR was calculated for the myocardium by dividing the amplitude of the ^23^Na resonance in the spectrum by the standard deviation of the real component of the noise between 10–20 ppm, distant from the ^23^Na resonance; the chemical shift of the ^23^Na resonance was defined as 0 ppm.

## Results

**Fig. 2:**
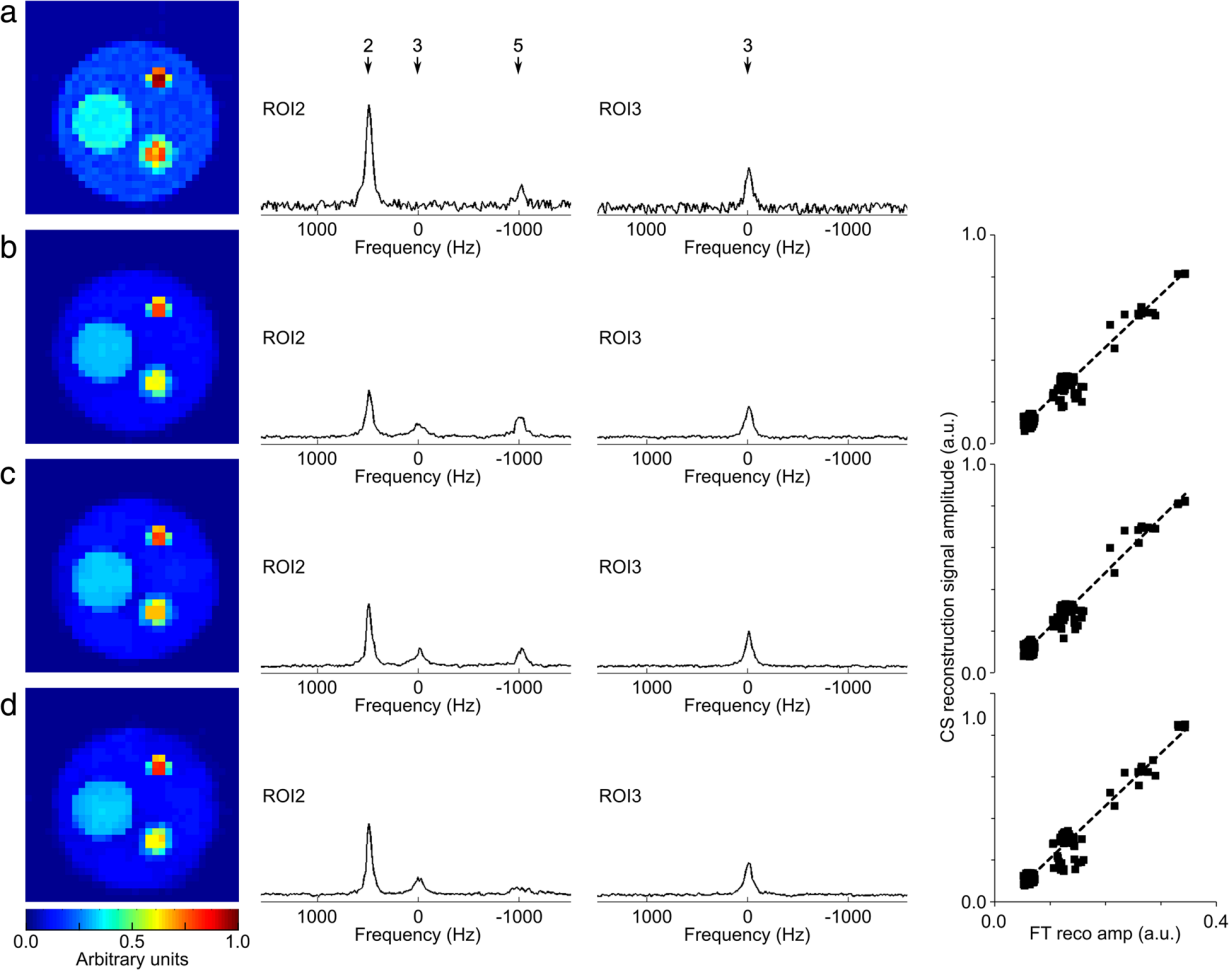
Example reconstruction of virtual phantom. Normalized signal amplitude maps (PE = 32, SNR = 32) are shown in the left column for (**a**) FT reconstructed fully-sampled data, and CS reconstructed data for (**b**) R = 1, (**c**) R = 3, and (**d**) R = 5. Example spectra from ROI2 and ROI3 are shown in the middle two columns. The ROI from which each resonance originates is indicated above the spectra. Due to blurring of compartment boundaries, there is signal contamination from compartments 3 and 5 evident in the spectrum for compartment 2; likewise the signal amplitude of the peaks in compartment 2 is reduced relative to the FT data. Note the denoising effect of the CS reconstruction as acceleration factor increases. All images and all spectra are plotted on the same scale. Correlation plots of FT reconstructed noise-free signal amplitudes against raw CS reconstructed amplitudes (i.e. not normalized) for the same data are depicted in the right column for R = 1, 3 and 5. Linear regression yielded slopes of 2.52, 2.61 and 2.53, with R^2^ values of 0.96, 0.96, and 0.94, respectively. All correlation plots share the same horizontal axis

Typical CS reconstructed data from the virtual phantom (SNR = 32, 32 PE steps) are shown in Fig. 2, demonstrating similar spatial and relative signal intensity distribution to the FT data. Consistent with previous studies [13], low SNR (i.e. ≤ 8) resulted in an exaggerated noise floor in the CS reconstructed data preventing the reconstruction from converging; these data were excluded from further analysis. Although absolute signal intensities relative to the fully-sampled noise-free FT reconstructed case were not preserved, CS reconstruction resulted in a linear scaling of the data (right column in Fig. 2). Importantly, all CS reconstructed data demonstrated a high correlation with FT reconstructed noise-free fully-sampled data, with gradients ranging from 1.30 (R = 4, PE = 16, SNR = 16) to 5.73 (R = 1, PE = 64, SNR = 32), R^2^ values from 0.80 (R = 4, PE = 16, SNR = 16) to 0.98 (R = 1, PE = 64, SNR = ∞), and *p*-value always <0.001.

**Fig. 3:**
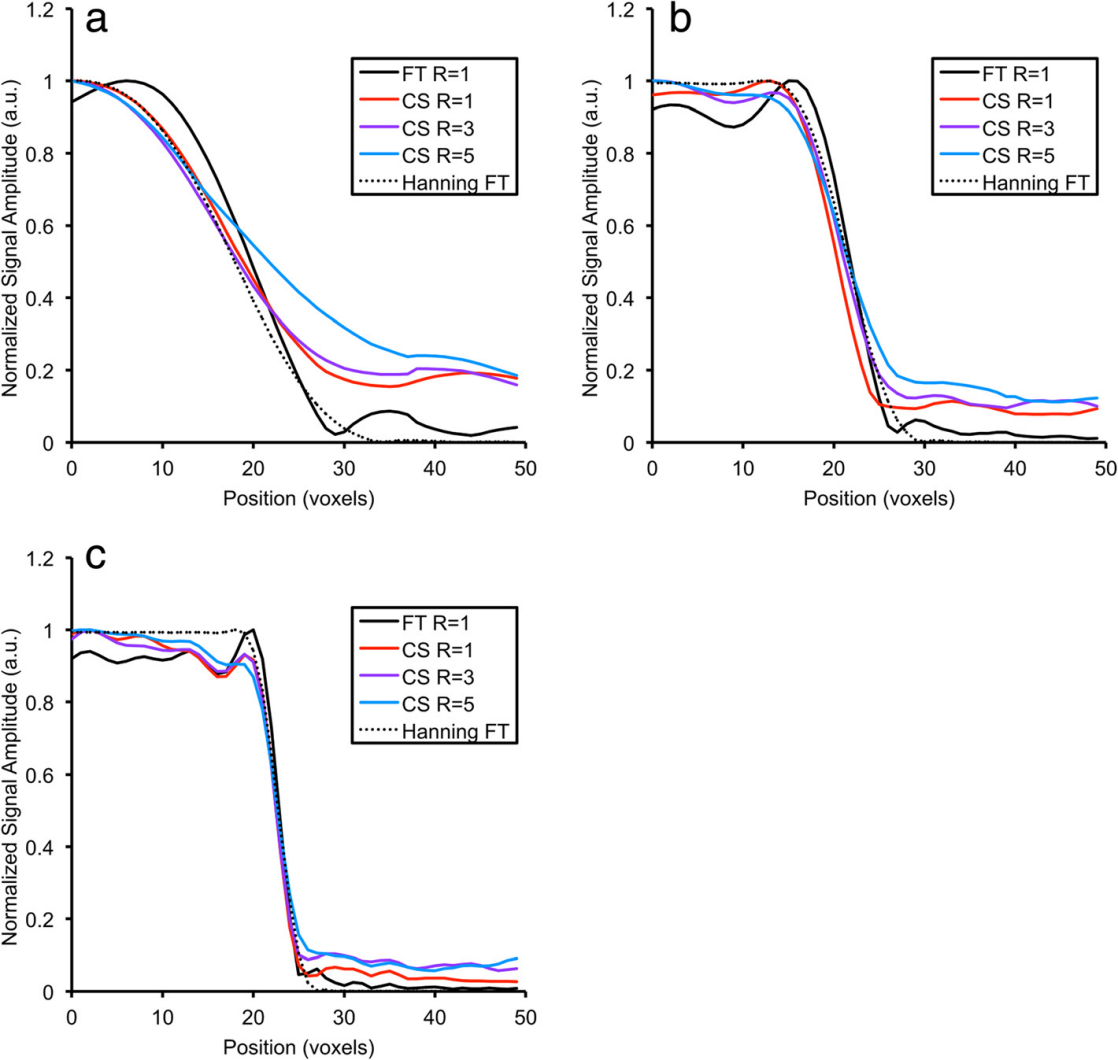
Effect on spatial resolution of CS reconstruction. Mean signal profiles at the boundary of compartment 3 of the virtual phantom as an illustration of the point-spread function using (**a**) 16, (**b**) 32, and (**c**) 64 phase encoding steps are shown for FT reconstruction (bold black line) and CS reconstructions with R = 1, 3 and 5 (red, purple, and blue lines respectively). The profile from equivalent acquisition-weighted (Hanning) FT reconstructed MRSI data is included for comparison (dotted line). All data are normalized to a maximum amplitude of 1.0

Higher acceleration factor resulted in denoising of the reconstructed spectra, but also in increased blurring of compartment boundaries, particularly at lower resolutions. This is qualitatively illustrated by the boundary of ROI3 (Fig. 3). While the FT reconstruction of the fully sampled data yielded the sharpest transition from signal within ROI3 to the outside, oscillations in signal amplitude outside the compartment are visible as a result of the point-spread-function of the uniformly weighted acquisition. Conversely, CS reconstructions of (R = 1 or 3) reduced these oscillations at the boundary transition, but resulted in an increased near-constant signal-floor outside the compartment, which decreased with improving spatial resolution.

**Fig. 4:**
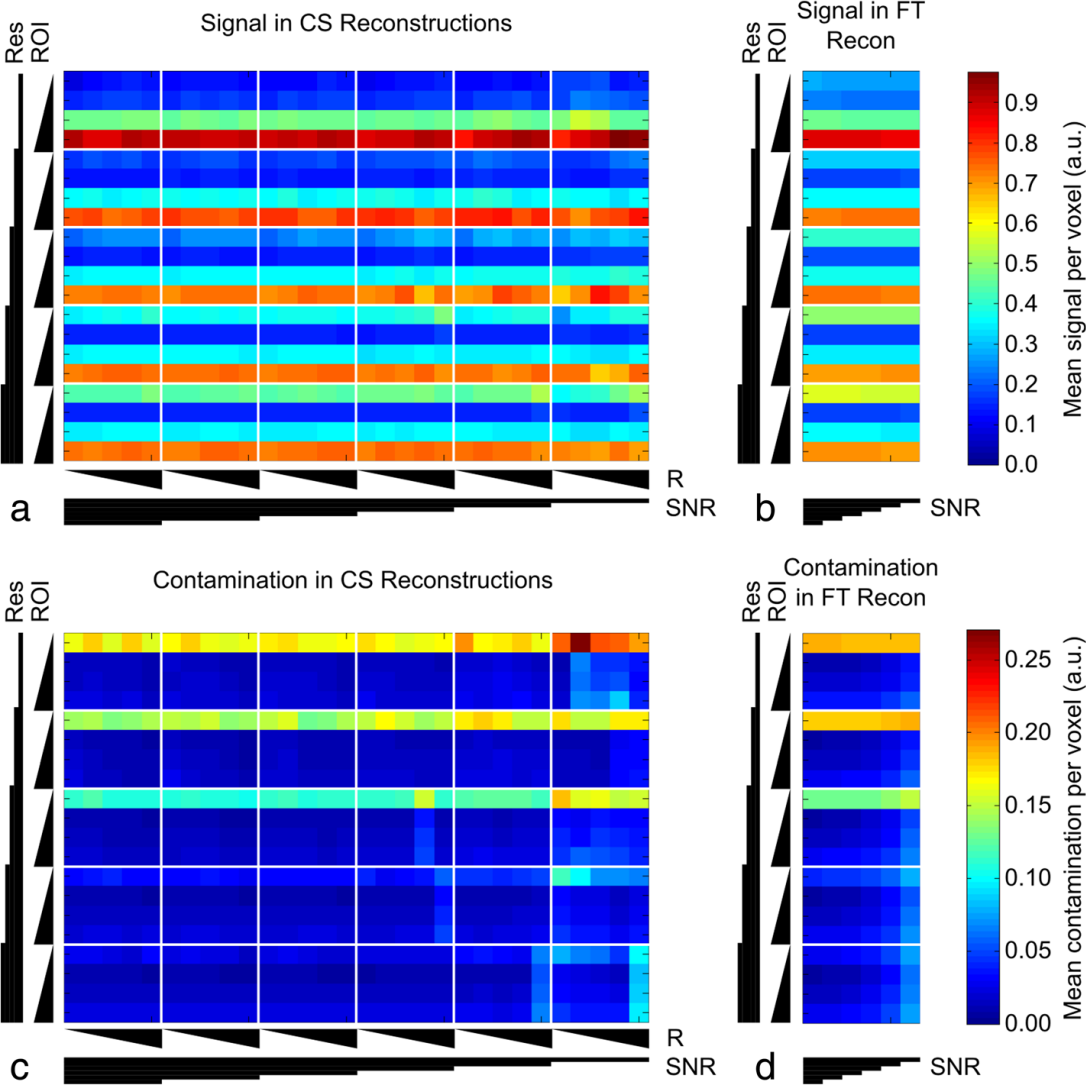
Signal amplitude and contamination for virtual phantom reconstructions. Mean signal amplitude from within an ROI normalized to the mean signal of ROI1 is plotted against number of phase encoding steps (Res; PE = 16, 24, 32, 48, 64), ROI number (ROI; 2–5), acceleration factor (R; 1–5), and SNR (∞, 256, 128, 64, 32, and 16) for (**a**) CS and (**b**) FT reconstructions; plots share the same scale. The CS plot (**a**) is divided into 5×4 element rectangles, each corresponding to CS reconstructions at a given SNR/spatial resolution. Columns correspond to acceleration factor (R = 1-5; left to right) and rows to ROI2-5 (top to bottom). SNR decreases towards the right and resolution increases towards the bottom. The FT plot (**b**) is divided into 6×4 element rectangles, each rectangle corresponding to FT reconstructions carried out at a given spatial resolution; columns correspond to SNR (∞, 256, 128, 64, 32, and 16; left to right) and rows to ROI2-5 (top to bottom). The number of phase encoding steps increases towards the bottom of the plot. Triangles below/left of the plots indicate parameter magnitude with the thin end corresponding to the lowest value. If a CS reconstruction performs identically to the equivalent FT reconstruction, then the column of the CS plot (**a**) should appear identical to the corresponding column of the FT plot (**b**). Mean signal contamination arising from outside an ROI detected within that ROI normalized to the mean signal from ROI1 is plotted against number of phase encoding steps, ROI number, CS acceleration factor, and SNR for both (**c**) CS and (**d**) FT reconstructions; plots share the same scale. Interpretation of the plots is analogous to that of the amplitude plots above. Increasing SNR and decreasing acceleration factor improves agreement between CS and FT reconstructions. Note the separate scale bars for amplitude and contamination plots

Figure 4 shows the color-coded (a,b; top row) signal amplitude and (c,d; bottom row) contamination for ROI2-4 of the virtual phantom, normalized to the mean signal intensity from ROI1. Data were normalized to the signal from ROI1 as this compartment provided a defined signal and had dimensions similar to those that would be expected from a concentration reference phantom that could reasonably used in murine cardiac *in vivo* experiments. Data are presented as a function of spatial resolution, acceleration factor, and SNR for the CS- (a, c; left column) and the FT-reconstructions (b, d; right column). Unsurprisingly, the highest contamination, and correspondingly lower signal amplitudes were found for the CS reconstructed data of ROI2 (representing left-ventricular myocardium) at low resolution. Similarly, low amplitudes and high contamination were found for CS reconstructions of all ROIs at lowest SNR and increasing under-sampling factors. Increasing SNR and resolution resulted in CS reconstructions that more closely resembled the FT reconstruction of fully sampled data, while contamination decreased to levels lower than in those for the FT reconstruction.

**Fig. 5:**
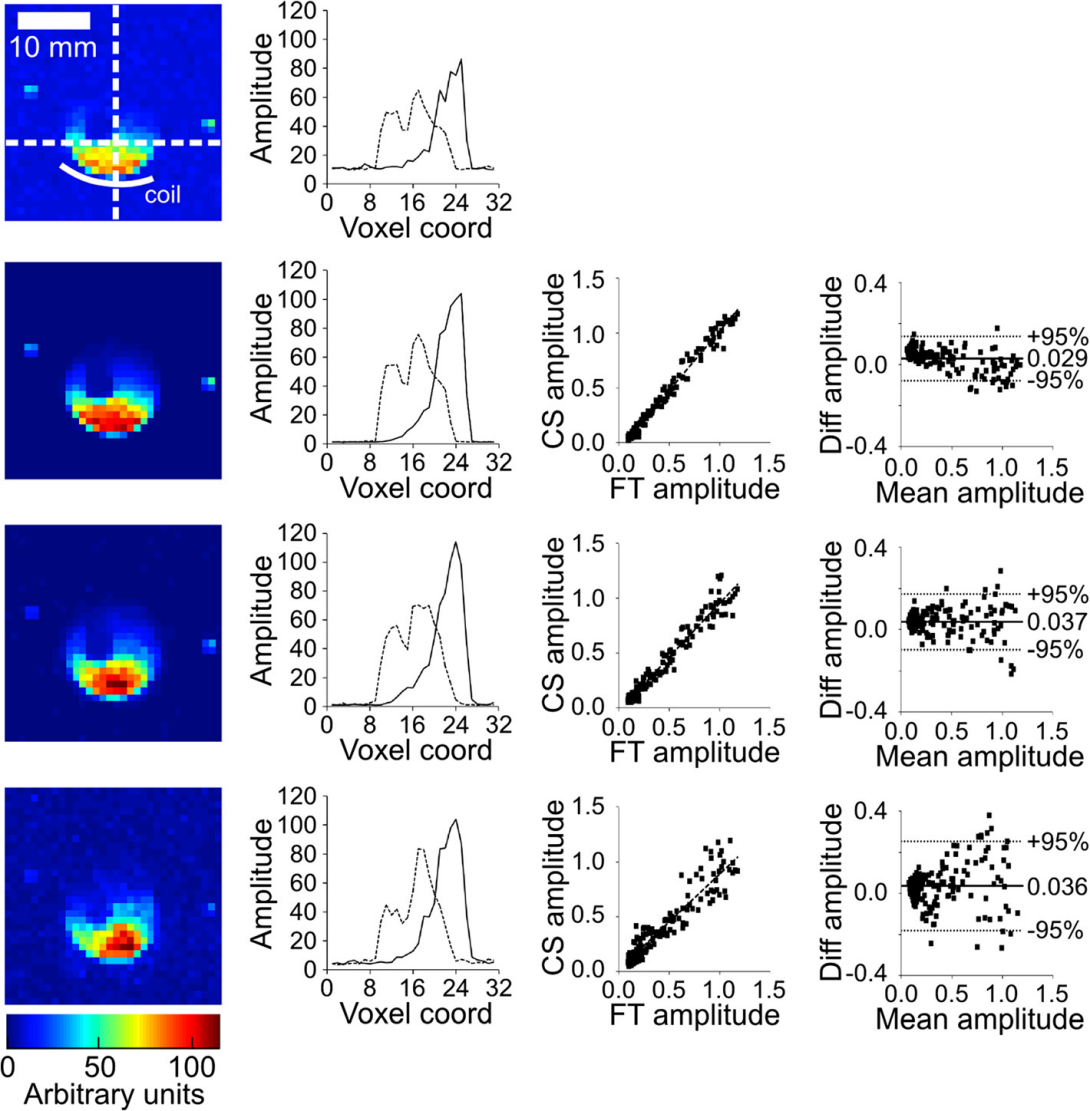
CS reconstruction of retrospectively under-sampled phantom data. Results of CS reconstruction of retrospectively under-sampled phantom CSI data acquired with one average and 32 × 32 PE steps. Images represent the signal amplitude (magnitude-modulated by the receive profile of the surface coil, the position of which is indicated in the top left panel) following FT reconstruction of fully-sampled data, and CS reconstruction of data with R = 1, 3, and 5 (top to bottom). All images are plotted on the same scale. The white dashed lines in the top left panel illustrate the position of the displayed profiles (second column from left); solid and dashed lines indicate vertical and horizontal profiles, respectively. Note the reduction in noise around the phantom arising from the CS reconstruction for R = 1 relative to the FT reconstruction and smoother profiles across the phantom. Increasing acceleration factor and decreasing SNR resulted in blurring of the boundaries of the phantom in the images. The third column shows the correlation between the normalized signal amplitude of fully-sampled FT reconstructed data (*x*-axis) and of the CS reconstructed under-sampled data (*y*-axis); data were normalized to the mean signal arising from the ROI indicated in Fig. 1c. Linear regression yielded slopes of 1.09, 0.98, and 0.88, with R^2^ values of 0.98, 0.96, and 0.89, respectively. The right-hand column shows Bland-Altman plots for normalized FT- and CS-reconstructed signal amplitudes. The mean difference in signal amplitude is indicated by a solid line, and 95 % confidence interval by dashed lines. The value of the mean difference in amplitude is indicated on the plots. All plot amplitudes are in arbitrary units

In Fig. 5, sodium intensity maps from the compartmented phantom are shown for FT reconstructed fully-sampled, and CS-reconstructed data (R = 1, 3 and 5; 32 PE steps). The gradient in signal intensities along the vertical axis of the images reflects the receive profile of the surface coil. Horizontal and vertical profile, correlation plots (normalized FT vs. CS reconstructed data), and Bland-Altman analysis for the amplitudes confirmed the behaviour observed in the virtual phantom: CS reconstruction resulted in a high correlation between CS and FT reconstructed data, in signal denoising, and in increased blurring of compartment boundaries with increased under-sampling.

**Fig. 6:**
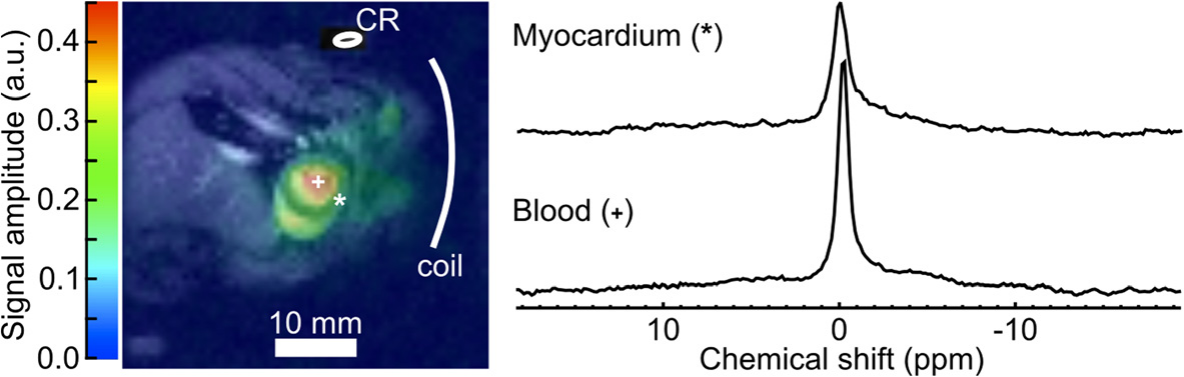
Representative *in vivo* CS three-fold accelerated cardiac MRSI. CS reconstructed prospectively under-sampled ^23^Na CSI (R = 3) of a murine heart in short-axis orientation (colour) overlaid onto a ^1^H anatomical image (black and white). The position of the surface coil is indicated. The concentration reference phantom (CR) has been omitted from the CSI data. The difference in Na concentration between the myocardium and blood pool is clearly reflected in the color-coded signal amplitudes of the ^23^Na image. Example phased spectra for the myocardium (*) and left ventricular blood (+) are shown on the right. Both spectra are plotted on the same scale

A prospectively 3-fold under-sampled, CS-reconstructed, myocardial ^23^Na-map in short-axis orientation obtained *in vivo* from a mouse overlaid on an anatomical ^1^H-image, and corresponding spectra, are presented in Fig. 6. Blood/myocardium amplitude ratios of 0.45 ± 0.07, 0.48 ± 0.09, and 0.47 ± 0.08 were obtained *in vivo*; assuming a blood ^23^Na concentration of ~79 mM [23], myocardial ^23^Na concentrations of 35.6, 37.9, and 37.1 mM can be estimated for the three experiments. The SNR in the anterior myocardium (close to the surface coil) was 42.1 ± 1.1, 49.2 ± 1.4, and 75.1 ± 1.8 (mean ± SD) whilst in the posterior myocardium (distant from the surface coil) it was 36.6 ± 1.3, 36.5 ± 2.4, and 61.2 ± 1.5 (mean ± SD), respectively.

## Discussion and conclusions

MRS(I) provides a unique method for investigation of metabolic processes in living tissue and has been successfully applied in clinical studies of brain and cancer metabolism (e.g. [24-26]). Specifically, ^23^Na-MRSI has the potential to monitor myocardial ion homeostasis in models of cardiac disease. Widespread application of MRSI remains limited, especially in the clinic, due to significant technical challenges, low sensitivity and associated long scan-times. A reduction in acquisition time without sacrificing accuracy would represent an important step towards making MRSI more widely applicable. The CS algorithm used in this work has been applied previously to study brain and prostate tumours with ^1^H-MRSI [11], however our study offers several novel aspects. By providing a thorough validation of the reconstruction, we sought to determine the (pre-) clinical applicability of CS-accelerated MRSI. The use of synthetic data ensured that the results were generally applicable, independent of organ/tissue and nucleus. It also permitted an assessment of cross-compartment signal contamination, which is not possible with (noise-free model of) clinical data where compartments share similar metabolite populations. To the best of our knowledge, only one study has reported the application of ^23^Na-MRSI to murine hearts *in vivo* [27]. ^23^Na-MR spectroscopy commonly utilises only a single resonance arising from both intra- and extra-cellular sodium, which may therefore also be detected with MR imaging. However, we used this nucleus to prove the concept of the technique, with the ultimate aim of combining it with multiple-quantum filters to distinguish intra- from extra-cellular sodium. Thus, application of CS to ^23^Na-MRSI could significantly reduce scan-time making metabolic data acquisition possible within clinically acceptable times.

There are several technical challenges to be faced in applying CS to ^23^Na-MRSI of (murine) cardiac tissue: large sodium concentration differences between tissues, and thus MRSI signal intensity variations, lie in close proximity (e.g. blood vs. myocardium vs. lungs), which can be difficult to reconstruct using CS; the influence of SNR and spatial resolution on CS reconstruction fidelity had to be characterized.

Virtual phantom data, which mimicked the appearance and challenges of an *in vivo* cross-section through a murine thorax, was used to characterize CS performance. For ROI2 (left-ventricular myocardium) this resulted in a wall thickness of between 1 and 3 voxels corresponding to ~0.5-1.5 mm in typical *in vivo* mouse cardiac MRSI experiment. We found that absolute signal amplitudes following CS reconstruction were not preserved, but correlated linearly with those obtained in the (noise-free) conventional FT reconstruction (i.e. “ground truth”). This finding has two immediate implications. Firstly, linear scaling of the amplitude means relative amplitudes (i.e. metabolite ratios) remain unaffected by CS reconstruction. Consequently, absolute quantification of metabolite concentration relative to a signal from a compartment of known concentration remains accurate. Secondly, the calculation of root-mean-square errors, which are typically used as a measure of data fidelity, would yield high values and not reflect the quality of the reconstruction. Thus reconstructed signal amplitudes and signal contamination, normalized to the mean signal intensity from a defined ROI, were chosen as metrics for this purpose (Fig. 4). For the CS reconstructions, increasing spatial resolution reduced signal contamination (bottom row, Fig. 4) and blurring of compartment boundaries (Fig. 3). Low-resolution FT reconstructed data yielded signal oscillations inside and outside the compartment due to convolution of the image with the FT of a step function. CS reconstruction reduced these oscillations at the cost of near-constant signal level outside the compartment, which increased with higher under-sampling factors and/or decreasing resolution. Whilst there is a straightforward relationship between data sampling and point spread function for FT reconstruction, the relationship is more complex for CS. The CS reconstruction used here assumes that an image consists of regions of approximately homogeneous signal with relatively sharp discontinuities in signal intensity between regions, and that the resulting reconstruction must be consistent with the acquired k-space data. These assumptions give rise to some blurring of the boundaries between homogeneous regions resulting in an apparent loss of spatial resolution and out-of-voxel signal contamination. Some understanding of the origins of a signal can be inferred from the spatial distribution of the reconstructed signal across a sharp boundary, for example at the edge of a phantom where the signal transitions from that originating from within the phantom to the void surrounding the phantom. Signal present in the void surrounding the phantom indicates of out-of-voxel contamination present in that voxel; Fig. 3 demonstrates this effect of CS and reveals that the out-of-voxel contamination approaches that present in FT reconstructed data as spatial resolution increases. CS can result in low-level signal contamination arising from signals a considerable distance outside the target voxel.

Our findings suggest in agreement with the literature [11], that up to 5-fold under-sampling of (pre-)clinical MRSI will be possible without significant impact on data fidelity given modest SNR. This conclusion was subsequently confirmed in retrospectively under-sampled phantom experiments. Prospectively accelerated *in vivo* data were also acquired. Notably, our murine study was at the lower end of the SNR requirements due to the very high spatial resolution; Neuberger et al. report that a voxel volume of 1 mm^3^ could be achieved within ~90 mins [27], which was over twice the volume used in our study. Thus, a conservative under-sampling factor of 3 was chosen *in vivo*. Additionally, the reconstruction of the phantom and *in vivo* data demonstrated that CS accurately reproduces spatially inhomogeneous signals arising from the receive profile of the surface coil used to acquire the data. The quantified myocardium/blood amplitude ratios agreed well with previously reported values of 0.42 ± 0.15 for mouse [27] and 0.57 for humans [23]. A 3-fold reduction in scan time to ~23 mins for a single slice makes our protocol suitable for use as part of a multi-parametric MR-phenotyping investigation, or to study severely sick animals by reducing the anaesthetic burden. Assuming that the typical mouse heart is approximately 8 mm in length, full heart coverage using 2D CS-MRSI as described would therefore take ~92 mins; this represents half the voxel volume described by Neuberger et al. within the same scan time. Furthermore, use of through-plane sparsity information in the CS reconstruction should allow further acceleration of 2D multislice or 3D CS-MRSI without loss of reconstruction fidelity. Compressed sensing has undoubtedly a significant role to play in the future of preclinical and clinical MRSI. Increases in total tissue sodium concentration of between 150 and 330 % have been observed for a variety of pathologies [28-31]. Fig. 3 would suggest that for resolutions at or above 32 × 32 PE steps, the level of potential signal contamination in a voxel arising from CS reconstruction would have an approximate upper limit of 20 %. However, in the presence of this level of signal contamination, such pathologies should be detectable. In general, the SNR of the data will fundamentally limit the ability to detect pathological abnormalities; whilst FT performs better than CS at very low SNR, accurate data fitting/quantification under these conditions may prove limiting even for FT reconstruction. Whilst the experimental results presented here focus on MRSI and its application to ^23^Na, the simulation results apply equally well to ^1^H, ^31^P, or ^13^C MRSI. Prospectively under-sampling data can significantly reduce MRSI data acquisition to a clinically acceptable time, reducing the burden on the patient and increasing scanner throughput without sacrificing diagnostic accuracy.

## Abbreviations

MRSI: Magnetic resonance spectroscopic imaging
CS: Compressed sensing
SNR: Signal-to-noise ratio
FT: Fourier-transform
EPSI: Echo-planar spectroscopic imaging
CSI: Chemical shift imaging
R: Acceleration/undersampling factor
